# 1243. Acceptable susceptibility thresholds reported in hospital antibiograms for Gram-negative rod infections: a survey and contingent valuation study of infectious diseases providers

**DOI:** 10.1093/ofid/ofad500.1083

**Published:** 2023-11-27

**Authors:** Shinya Hasegawa, Eli N Perencevich, Kimberly Dukes, Michihiko Goto

**Affiliations:** University of Iowa Carver College of Medicine, Iowa City, Iowa; University of Iowa/Iowa City VAMC, Iowa City, Iowa; University of Iowa Carver College of Medicine, Iowa City, Iowa; University of Iowa/Iowa City VAMC, Iowa City, Iowa

## Abstract

**Background:**

Clinical guidelines recommend an antibiogram as a key component when making empiric therapy decisions. However, little is known about how clinicians utilize antibiograms. We aimed to assess the interpretation thresholds of hospital antibiograms among infectious diseases (ID) providers when making empiric therapy decisions for Gram-negative rods (GNRs) infections.

**Figure 1.**
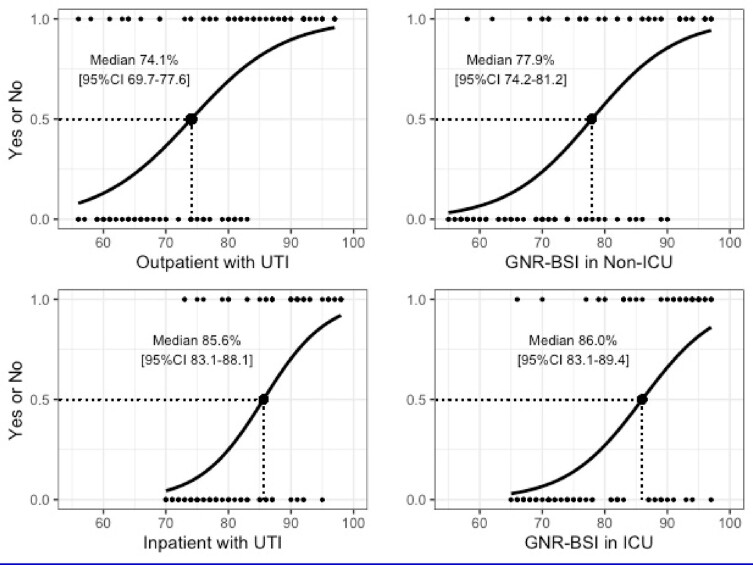
Relationship between randomly generated percentages on antibiograms in each scenario and the distribution of participants willing to accept the threshold. Abbreviations: GNR-BSI, Gram-negative rod bloodstream infection; ICU, intensive care unit; UTI, urinary tract infection.

**Methods:**

We conducted an email-based survey of ID providers practicing at Veterans Health Administration (VHA) facilities. We included four scenarios: i) a patient with urinary tract infection (UTI) in an outpatient setting, ii) a patient with UTI in an inpatient setting, iii) a patient with GNR bloodstream infection (GNR-BSI) in a non-intensive care unit (ICU) setting, and iv) a patient with GNR-BSI in an ICU setting. Each scenario randomly assigned antibiogram percentages and asked providers if they would feel comfortable selecting a hypothetical empiric therapy agent with the given value. Contingent valuation analyses were done by logistic regression models to evaluate the relationship between the percentages offered to providers and their willingness to use offered agents.

**Results:**

In the preliminary analysis, 112 of 599 providers (18.7%) responded and 104 provided effective responses. Hospital antibiograms are generally used infrequently, and only 30.8% of providers indicated that they use antibiograms more than once a month. The estimated median interpretation thresholds, meaning half of the ID providers would prescribe the hypothetical antibiotic in each case with the values given on antibiograms, were significantly higher for patients with more severe illnesses (85.6% [95% confidence interval [CI] 83.1-88.1] for an inpatient with UTI vs 74.1% [95%CI 69.7-77.6] for an outpatient with UTI; 86.0% [95%CI 83.1-89.4] for a patient with GNR-BSI in ICU vs 77.9% [95%CI 74.2-81.2] for a patient with GNR-BSI in non-ICU) (Figure 1).

**Conclusion:**

This study demonstrated that ID providers rarely utilized antibiograms and the median thresholds for hospital antibiograms influencing empiric antibiotic selection ranged from 74-86%, depending on the severity but not by the type of infection (UTI vs. BSI). Further analyses after we receive more responses will be completed.

**Disclosures:**

**Michihiko Goto, MD MSCI**, Merck & Co.: Grant/Research Support

